# Spring warming in Yukon mountains is not amplified by the snow albedo feedback

**DOI:** 10.1038/s41598-018-27348-7

**Published:** 2018-06-13

**Authors:** Scott N. Williamson, Faron S. Anslow, Garry K. C. Clarke, John A. Gamon, Alexander H. Jarosch, David S. Hik

**Affiliations:** 1 Department of Biological Sciences, University of Alberta, Edmonton, Alberta T6G 2E9 Canada; 2 Department of Earth and Atmospheric Sciences, University of Alberta, Edmonton, Alberta T6G 2E3 Canada; 3 Pacific Climate Impacts Consortium, University of Victoria, Victoria, British Columbia V8W 3R4 Canada; 4 Department of Earth, Ocean and Atmospheric Sciences, University of British Columbia, Vancouver, V6T 1Z4 Canada; 5 Institute of Atmospheric and Cryospheric Sciences, University of Innsbruck, Innrain 52f, A-6020 Innsbruck, Austria

## Abstract

Decreasing spring snow cover may amplify Arctic warming through the snow albedo feedback. To examine the impact of snowmelt on increasing temperature we used a 5,000 m elevation gradient in Yukon, Canada, extending from valley-bottom conifer forests, through middle elevation tundra, to high elevation icefields, to compare validated downscaled reanalysis air temperature patterns across elevational bands characterized by different patterns of spring snowmelt. From 2000 to 2014 we observed surface warming of 0.01 °C/a·1,000 m in May (0.14 °C/a at 1,000 m to 0.19 °C/a at 5,000 m), and uniform cooling of 0.09 °C/a in June at all elevations. May temperature trends across elevationally dependent land cover types were highly correlated with each other despite large variations in albedo and snow cover trends. Furthermore, a clear dependency of infrared skin temperature on snow cover mediated albedo decline was observed in tundra, but this was insufficient to influence average diurnal air temperature. We observed negative June temperature trends which we attribute to increasing daytime cloud cover because albedo and snow cover trends were unchanging. We conclude that 8-day and monthly averaged Spring air temperature trends are responding to a synoptic external forcing that is much stronger than the snow albedo feedback in sub-Arctic mountains.

## Introduction

Current global warming trends appear to be amplified both in the Arctic^1^ and at high elevation^2^. The loss of snow cover has been linked to enhanced terrestrial warming in the Northern Hemisphere^3,4^ and the surface albedo feedback has been specifically identified as contributing to amplified warming^5,6^. In climate models, feedbacks are often quantified using the radiative kernel method^7^ which is the difference in radiative fluxes at the top of atmosphere (TOA) related to a change in the feedback variable, and is implemented as a partial derivative. The Surface Albedo Feedback (SAF) in particular is characterised as TOA net energy flux related to the albedo decrease caused by an increase in surface temperature^8,9^. However, it is difficult to isolate a surface albedo feedback effect related to snow cover decline at high latitude when external forcing simultaneously causes both snow cover decline and a surface feedback. This difficulty is further exacerbated by the coarse resolution of reanalysis data^10^ and also by the dependence of daytime surface temperature on cloud cover which modulates net radiation^11^ and is a driver of the snow albedo feedback. At present, there is no consensus regarding the strength of SAF in Arctic amplification from model responses to different forcing^12^ or between models and surface observations^8^.

Temperature reanalysis data, which generally is a homogenised regularly spaced gridded product that ingests observations from multiple instruments, has shown that Arctic amplification is heavily dependent upon the transport of heat from low latitudes^13^, a pattern deduced from observations of year-round warming of Arctic mid-troposphere, without coincident surface warming, as expected with SAF^1^. However, the mid-tropospheric warming signal found in the ERA-40 reanalysis product^13^ has been disputed because of heterogeneities in the reanalysis product^14–16^. Reanalysis products such as ERA-40 are typically produced at a spatial resolution on the order of 1° latitude and longitude, which can hinder the ability to resolve temperature changes related to surface processes, such as snow phenology, which are often constrained by elevation or land cover features varying at finer spatial scales. Furthermore, SAF is typically investigated using temporal averages of a month or longer which diminish the influence of surface processes operating on shorter time scales. These broad spatial and temporal resolutions cannot readily resolve the changes related to seasonal snow cover duration and extent occurring at finer scales, leaving underlying mechanisms unclear. Lastly, the use of binary snow cover classification, which provides the ability to extend trend analysis to the beginning of the satellite Earth observation record, further complicates the attribution of albedo changes to snow cover change. The estimated albedo feedback from the Northern Hemisphere cryosphere, attributed equally to reduced sea-ice and terrestrial snow cover using a binary snow cover classification, ranges from 0.3 to 1.1 W m^−2^ K^−1^ between 1979 and 2008^8^.

In the absence of surface albedo feedbacks in idealised models and under a doubling of atmospheric CO_2_, Arctic amplification is produced by the poleward movement of moisture and heat^17–19^. Indeed, experiments that neutralized SAF in 12 Intergovernmental Panel on Climate Change Fourth Assessment Report (AR4) models with regionally fitted zero-dimensional energy balance, found that SAF was a contributing, but not a dominating, factor in Arctic amplification^6^. The high-latitude responses in the multiple types of forcing between models are too broad to define the particular causes of Arctic temperature increase^12^. The 25 models in the Fifth Coupled Model Intercomparison Project (CMIP5) show a range of 0.03 to 0.16 W m^−2^ K^−1^ for SAF, with an average of 0.08 W m^−2^ K^−1^ ^9^, which is much lower than the estimates given above. The range indicated by Flanner *et al*.^8^ is larger than both the AR4 multi-model albedo feedback of 0.26 ± 0.08 W m^−2^ K^−1^ ^20^ and values calculated from 18 Third Coupled Model Intercomparison Project (CMIP3) models^8^. Investigation of CMIP5 models^5^ reveals that a warming surface radiates more energy when the surface is at a higher absolute temperature (a temperature feedback known as the Planck Feedback) and the contribution by the surface albedo feedback was less important in terms of its contribution to temperature change^5^. In the Arctic, the surface and the troposphere are decoupled through a positive lapse-rate feedback^5^, which amplifies surface warming compared to the upper troposphere. Furthermore, the Arctic and high elevation mountain regions are cold in absolute terms compared to low elevation middle latitudes. Consequently, an external forcing, such as heat transport from mid-latitudes, will appear to amplify warming through a Planck Feedback over the relatively cooler regions^5,21^. These contradictory hypotheses illustrate that the dominant causes of Arctic Amplification remain unresolved in some cases, even when we know the component mechanisms.

To address these issues, we investigated the effect of terrestrial snow cover and albedo reduction and external forcing in modulating May and June surface temperature in the southwest Yukon between 2000 and 2014. Primary data sources include high resolution dynamically downscaled North American Regional Reanalysis (NARR) air temperature and MODerate resolution Imaging Spectroradiometer (MODIS) satellite imagery of infrared Land Surface (skin) Temperature captured near solar noon (snLST), Fractional Snow Cover, daytime Cloud Cover, Broadband Black Sky Albedo (over vegetated surfaces) and Snow albedo (over permanent snow and ice. We use two measures of surface temperature, snLST and air temperature, because of the temporal scale differences in their response to synoptic conditions and net radiation^22^, and because they respond differently to snow fraction change^23^ especially in a mountainous environment^24^. To help resolve questions related to snow cover and albedo change as drivers of high latitude and elevation temperature change, we took advantage of the large elevation gradient and differential spring snow melt rates found around Mount Logan, Yukon, as a natural experiment to clarify the roles of snow-mediated albedo and daytime cloud cover changes in amplified warming. Specifically, the differential timing of spring snowmelt over the Mount Logan massif elevation gradient (approximately 5,000 m elevation change over 120 km) was used to separate the snow-mediated albedo decline caused by external forcing from that caused by the snow albedo feedback. The Conifer land cover, situated at the bottom of the elevation gradient was largely snow free during the study period, and acted as a reference standard when compared to the large snow cover changes occurring at higher elevation. We hypothesised that a decline in snow albedo and snow extent is the result of externally forced temperature increase and that the snow albedo feedback is of minor importance in recent amplified warming at high elevation in the Arctic. We predicted that any change in snow cover or snow albedo will have a measurable effect on solar noon LST, but these changes will not scale to surface air temperature because of the predominantly overriding effect of external forcing on air temperature.

## Geographical Context

**Figure 1:**
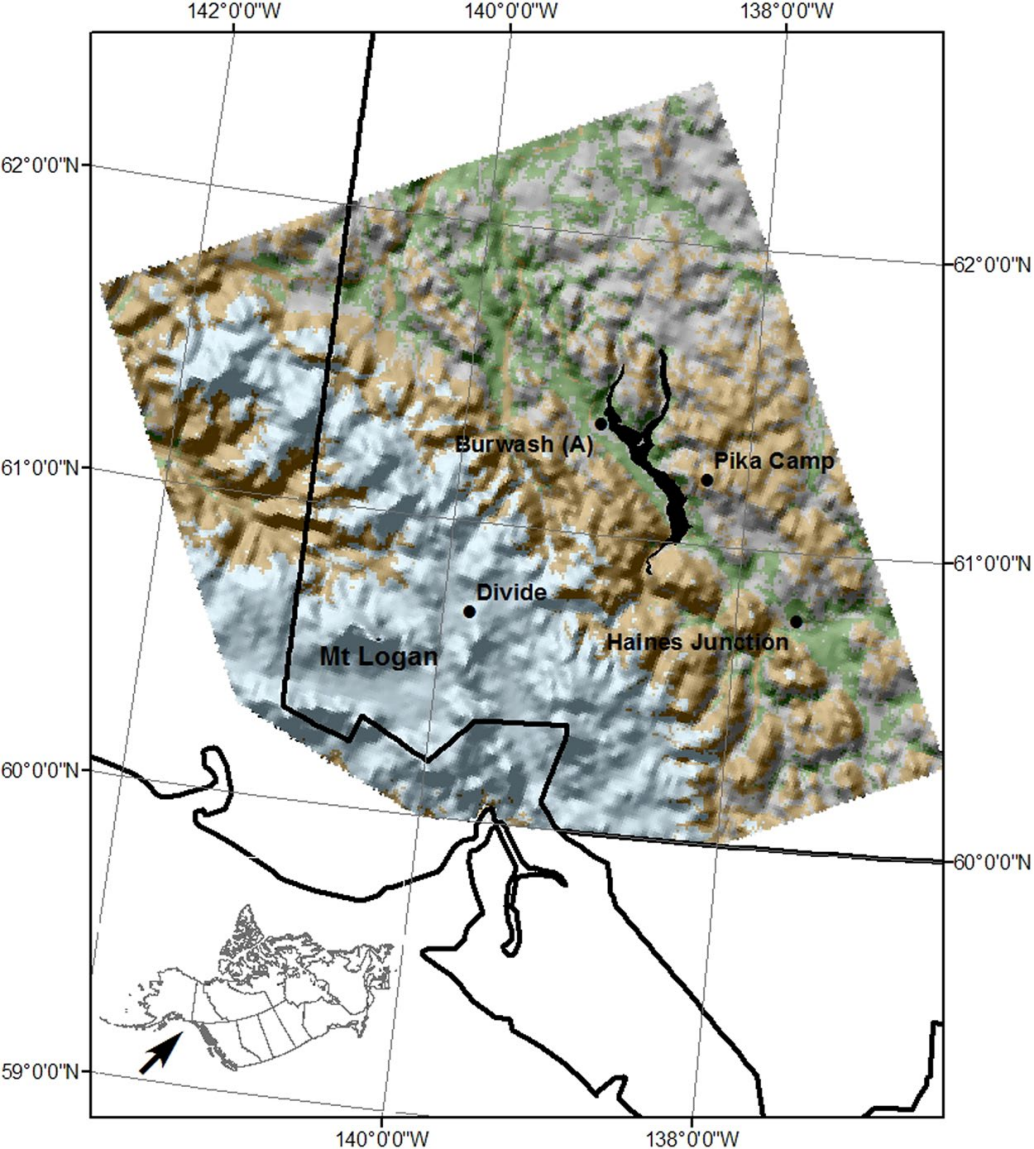
The Yukon and Alaska study area. Mount Logan (5,959 m) is located in the southwest corner of the Yukon Territory. The central dark dendritic feature is Kluane Lake (approximate elevation 781 m), the largest Lake in the Yukon. Light blue represents permanent snow and ice (Icefields), brown represents sparse vegetation (Tundra) which is located above tree-line, and green represents conifer forests comprised largely of white spruce (*Picea glauca*). Mixed land cover classes, including deciduous forest, found mostly on the northeastern portion of the study area, are coloured gray and have been removed from the analysis. The Environment Canada monitoring station at Haines Junction is found within the conifer land cover class. The Divide and Pika Camp meteorology stations provide temperature validation for Icefield and Tundra, respectively. Figure 1 was generated with ArcGIS 10.2.1 (www.esri.com).

The study area measures 59,503 km^2^ and is located north of 60° in the southwest Yukon, Canada and southeast Alaska and includes northern portion of the Alaska Panhandle (Fig. 1). This area includes many peaks of the St. Elias Mountains and Canada’s highest mountain, Mount Logan (5,959 m). The Mount Logan massif displays the largest base circumference of any non-volcanic mountain in North America and has eleven peaks over 5,000 m. The study design takes advantage of the lack of forest canopy cover over the majority of the study areas, which aids the precise identification of surface properties, such as snow cover and albedo, with remote sensing. We further reduce the potential for underestimating snow cover by using the GLOBCOVER land cover classification, produced by the European Space Agency, to reduce the land covers considered into high elevation permanent snow and ice (Icefield), mid-elevation sparse vegetation (Tundra), and low elevation needle leaved deciduous or evergreen forest (Conifer). The omitted classes contain tall statured vegetation that had the ability to mask snow cover. The tundra class can experience an increase in albedo during the growing season related to shrub canopy growth^25^, but this behaviour is negligible in comparison to the snow mediated albedo decline. These three classes are the most abundant in the study area with Conifer having 6,908 1 km^2^ grid cells, Tundra 18,468 and Icefield 19,969. The Icefield class has an average elevation of 2,230 m ± 1,227 m (95% Confidence), the Tundra class has an average elevation of 1,560 m ± 821 m and the Conifer class has an average elevation of 946 m ± 382 m. The spring snow cover has predominately melted by the beginning of May in the Conifer class. May and June snow cover extent in the Arctic has declined by 14% and 46%, with the southwest Yukon exhibiting a spring snow cover duration decline of approximately 6–8 days per decade between 1967 and 2008^26^.

## Results

**Figure 2:**
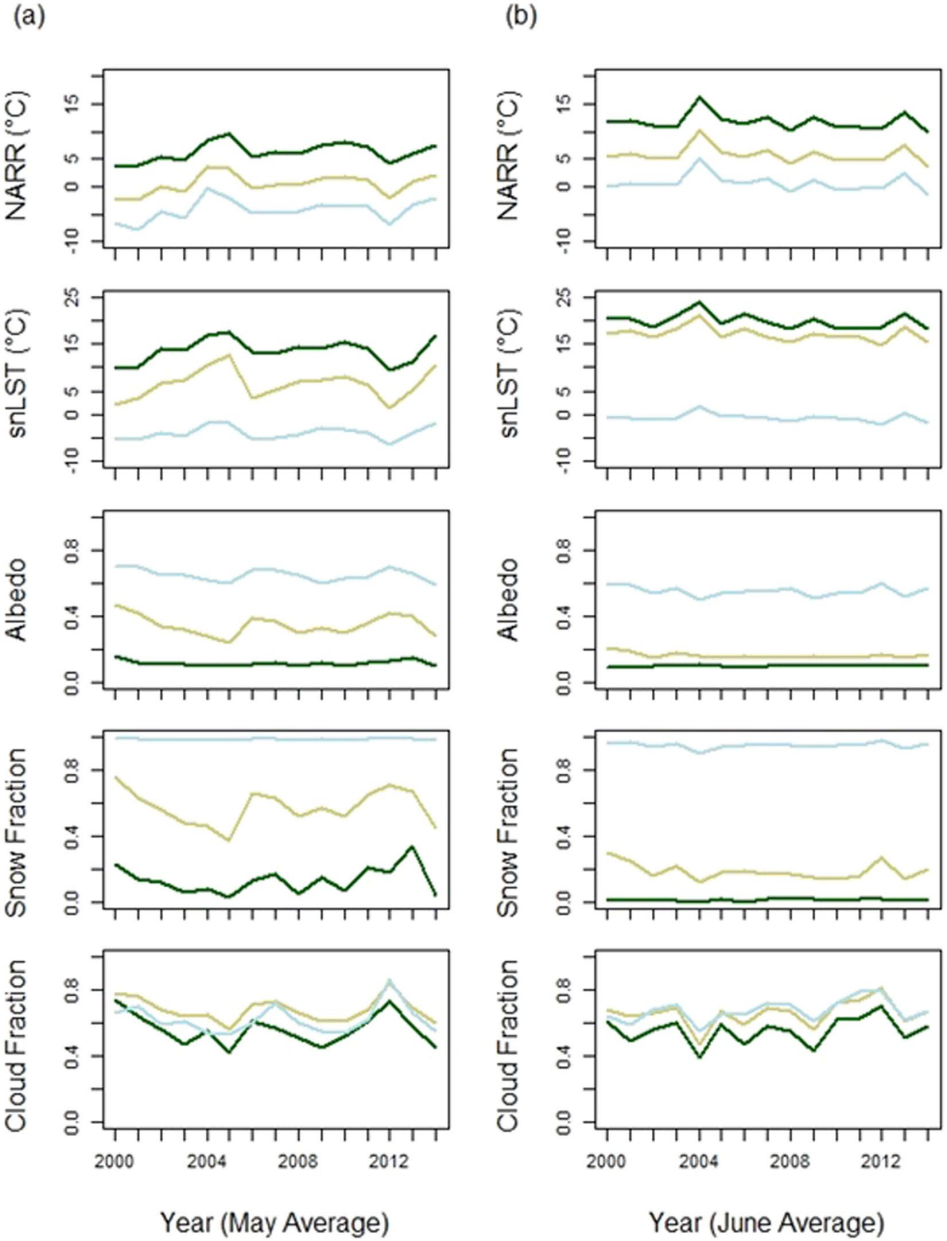
Time series of May (**a**) and June (**b**) monthly average downscaled NARR, MODIS solar noon LST, black sky albedo (Tundra and Conifer) and snow albedo (Icefield), snow fraction and daytime cloud fraction, plotted as monthly averages between 2000 and 2014. The low elevation Conifer class (dark green), Tundra class (brown) and Icefield (light blue) are plotted as the average values originating from all elevations these land covers occupy in the study area. Standard errors are small and have been removed for clarity. The mean values and standard errors used to produce the May and June plots are included as Supplementary Tables.

Time series of downscaled NARR, MODIS solar noon LST, black sky albedo (Tundra and Conifer) and snow albedo (Icefield), snow fraction and daytime cloud fraction are shown in Fig. 2 for the Conifer, Tundra and Icefield classes. Figure 2a displays May averages and Fig. 2b shows monthly averages for June. Green represents the average values for the five variables contained across all elevations within the Conifer class. Brown represents the Tundra class and light blue represents the Icefield class. The May and June NARR time series are highly correlated across different surface types despite large variations in snow fraction, albedo and MODIS solar noon LST, which strongly suggests that a synoptic external forcing is controlling air temperature. The correlation between May average NARR values between Tundra and Conifer is R^2^ = 0.94 and between Tundra and Icefield is R^2^ = 0.96. The correlation between June average NARR values between Tundra and Conifer is R^2^ = 0.99 and between Tundra and Icefield is R^2^ = 0.99. A very strong snow cover decline in May between 2000 and 2005 shows a strong influence on Tundra albedo and MODIS solar noon LST. In the Conifer land cover the NARR temperature increased by 5.9 °C, where in the Tundra class the increase was 5.3 °C. This occurred despite a solar noon LST increase of 7.7 °C in Conifer and a 10.4 °C increase in Tundra. The black sky albedo decreased in Conifer by 6%, where the Tundra albedo declined by 23%. This snow cover mediated albedo decline is considerably smaller in June than in May. These results indicate that snow cover decline does lead to an increase in absorbed solar radiation, which translated to a warmer surface, but this wurface warming does not scale to air temperature increase. The variability of snow cover for the Conifer class does not clearly influence the albedo for this class, which is likely because the snow cover is obscured by the conifer canopy. Cloud cover is also highly correlated between land covers, indicating that the variability in solar forcing is largely consistent between land covers.

**Figure 3:**
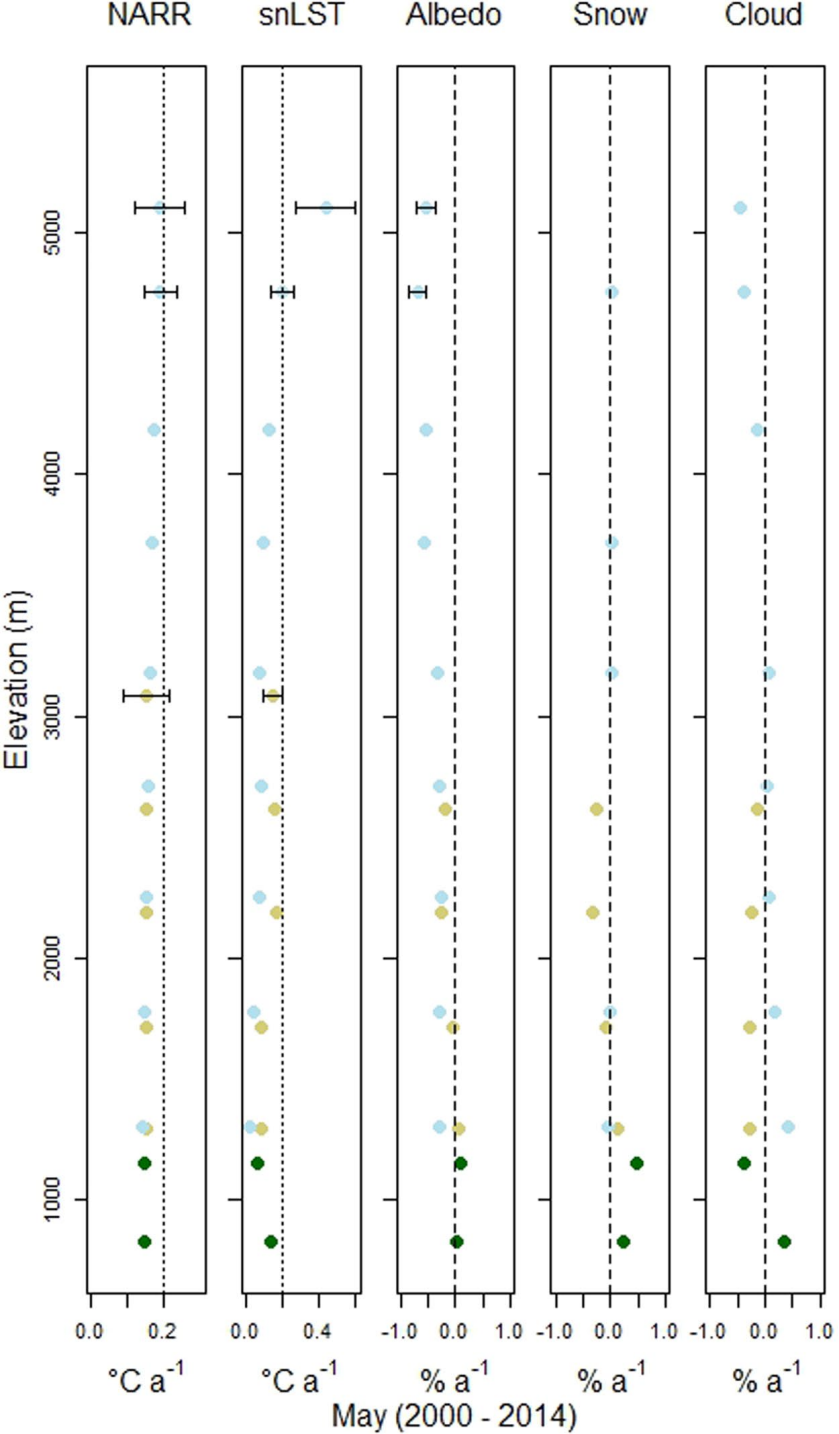
Points represent the linear slopes for the May trends of the five variables used in this study between 2000 and 2014. The light blue, brown, green points represent Icefields, Tundra and Conifer land covers, respectively. MODIS black sky albedo (MCD43A3) is used for Conifer and Tundra classes, and MODIS snow albedo (MOD10A1) is used for Icefields. All points are significant at p < 0.05. Standard errors are presented as error bars when larger than the point width. The points represent 500 m elevation bins, starting at 500 m and ending at 5,500 m. The dotted line is a 0.2 °C a^−1^ reference, where the dashed line separates positive from negative trends.

Figure 3 shows the statistically significant linear temporal trends (p-value < 0.05) for the five variables presented in Fig. 2a, separated into 500 m elevation bins between 500 m and 5,500 m, for the month of May between 2000 and 2014. Strong elevational dependence was found in the NARR and snLST trends, where the NARR warming rates increase linearly from 0.14 °C/a at 1000 m to 0.19 °C/a at 5,000 m. In the Icefield, the albedo trend was negative for all elevations and became strongly negative at higher elevation, which provided a mirror image of temperature. Albedo trends in Conifer were largely unchanged, and in Tundra were either unchanging or mildly negative. The daytime cloud cover trend became negative above 4,000 m, and was positive below 2,000 m.

**Figure 4:**
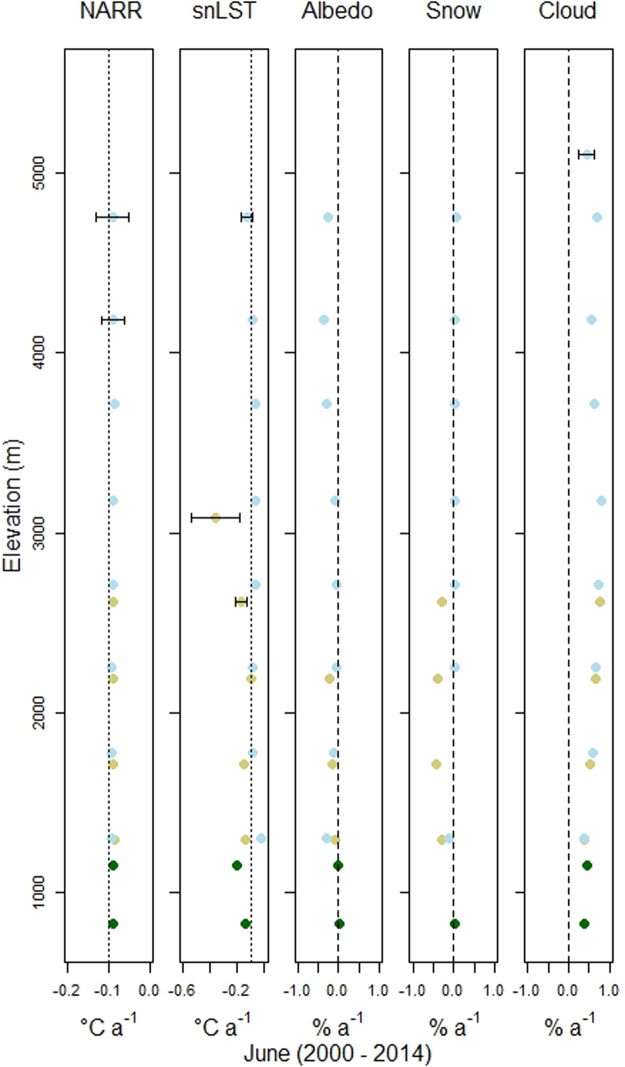
Points represent the linear slopes for the June trends of the five variables used in this study between 2000 and 2014. The light blue, brown, green points represent Icefields, Tundra and Conifer land covers, respectively. MODIS black sky albedo (MCD43A3) is used for Conifer and Tundra classes, and MODIS snow albedo (MOD10A1) is used for Icefields. All points are significant at p < 0.05. Standard errors are presented as error bars when larger than the point width. The points represent 500 m elevation bins, starting at 500 m and ending at 5,500 m. The dotted line is a −0.1 °C a^−1^ reference, where the dashed line separates positive from negative trends.

Figure 4 shows the statistically significant linear temporal trends (p-value < 0.05) for the five variables presented in Fig. 2b, separated into 500 m elevation bins between 500 m and 5,500 m, for the month of June between 2000 and 2014. The elevational dependence for NARR and snLST temporal trends in May are no longer present in June, with a −0.09 °C/a cooling trend in NARR temperatures uniformly expressed from below 1,000 m to 5,000 m. In Tundra, the snow cover and albedo trends are both negative and the albedo trend in Icefields becomes strongly negative above 3,500 m. There is no trend in Conifer albedo in either elevation bin in June. The daytime cloud cover temporal trends in June are almost uniformly positive throughout the elevation gradient. By contrast, in Tundra the trend in daytime cloud cover in May was negative over all elevations. The daytime cloud cover trend over the Conifer class was negative at the higher elevation but positive at lower elevation.

**Figure 5:**
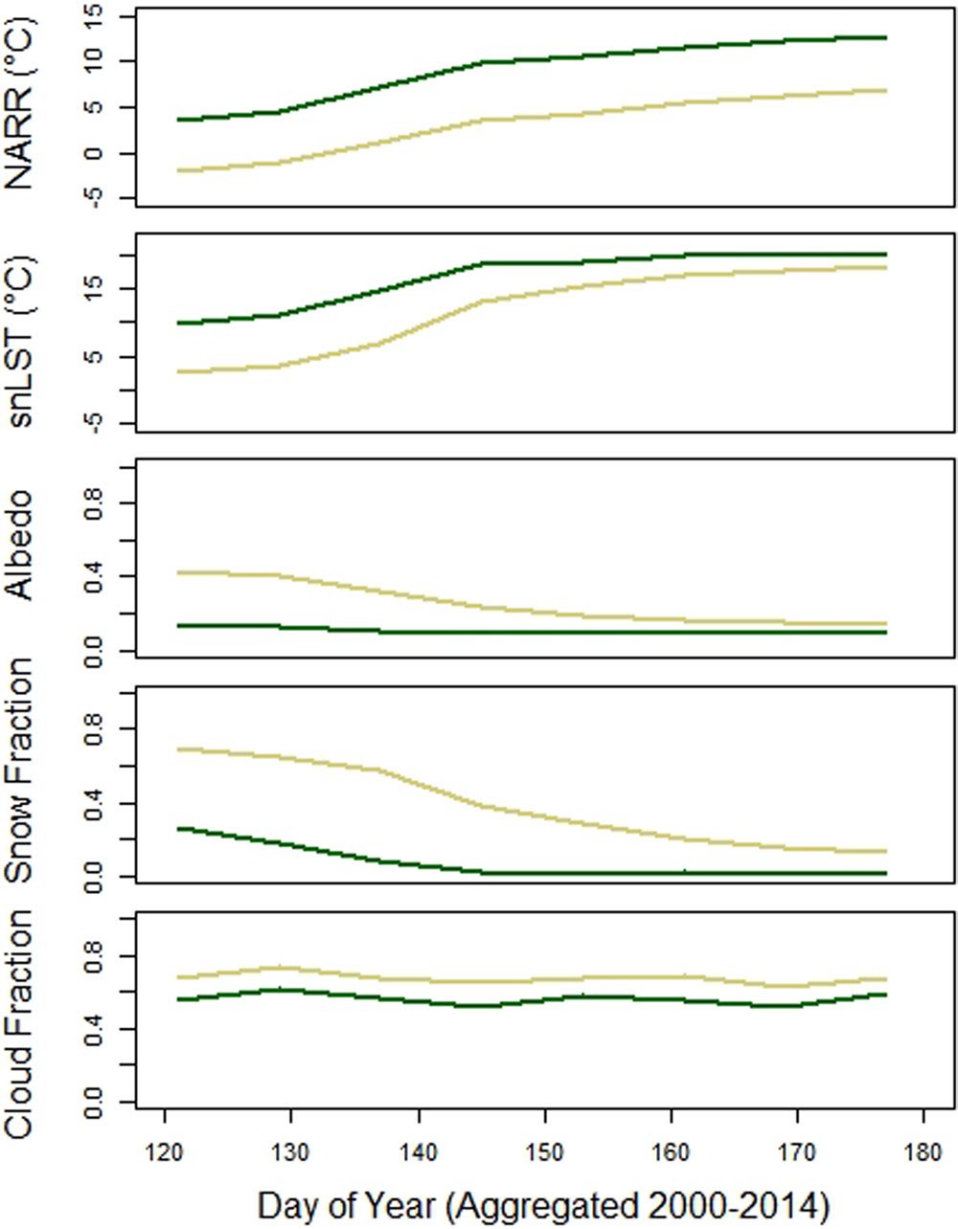
Seasonal phenology for Tundra (brown line) and Conifer (green) in key environmental variables from 2000 to 2014 data averaged by 8-day period. Standard errors are small (similar to line thickness) and have been removed for clarity.

Figure 5 displays the data from Figs 2 and 3 as 8-day averages composited over the entire 2000 to 2014 study period for the Conifer and Tundra land cover classes. Between classes, the downscaled NARR air temperature trends are highly correlated (R^2^ = 0.98), as are the day time cloud cover trends (R^2^ = 0.89). The differential effect from the seasonal snow cover decline on albedo and solar noon LST is clearly visible. The influence of the seasonal decline in snow cover is clearly seen for albedo and solar noon LST. Daytime cloud cover shows little variation between land cover types, beyond a consistently higher cloud fraction over Tundra. The downscaled NARR temperatures were highly correlated between Conifer and Tundra land covers, despite displaying differing snow cover, albedo and snLST time series.

### Validation

**Figure 6:**
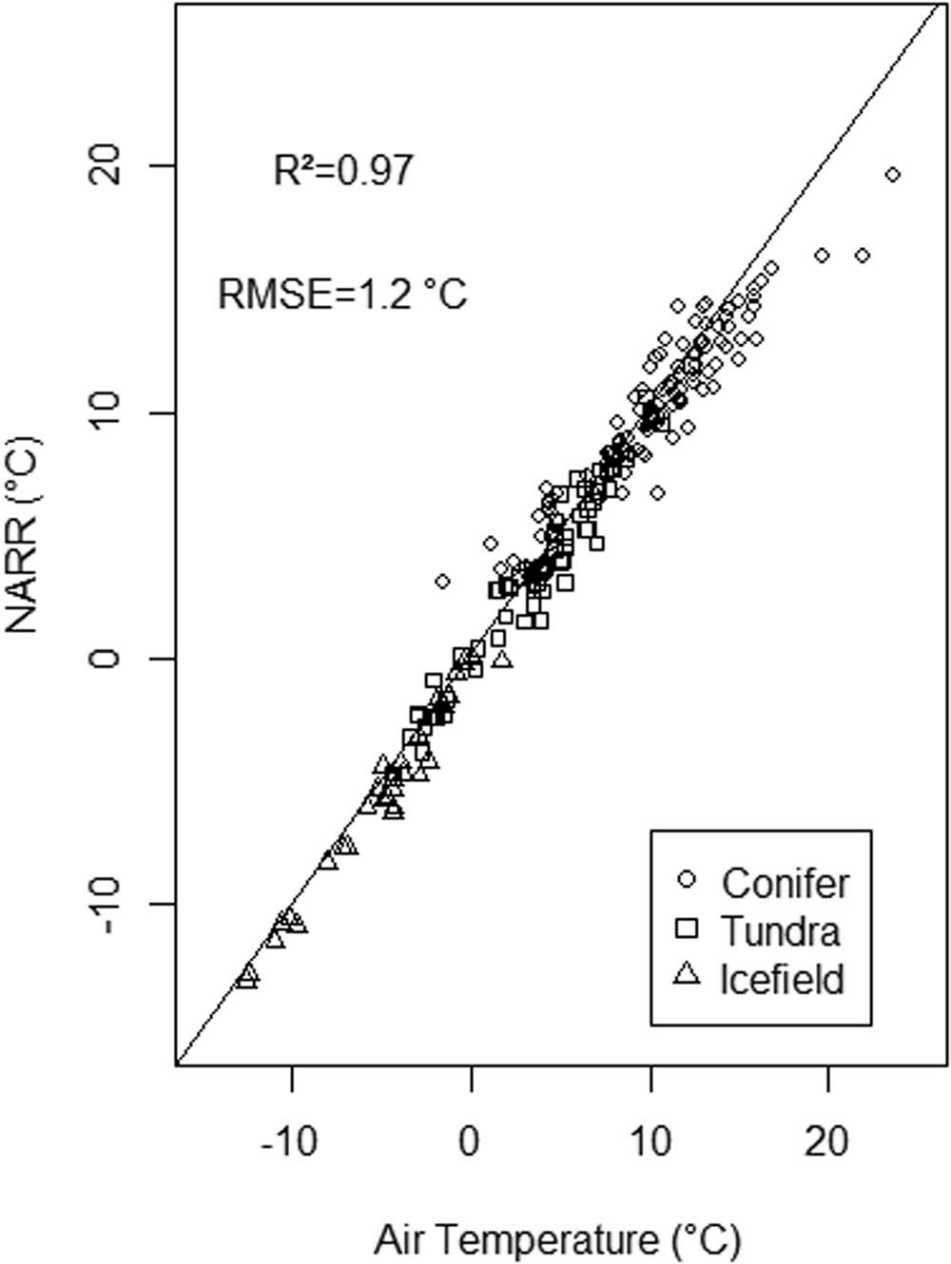
NARR downscaled 8-day temperature validation using meteorological measurements of 8-day average air temperature, for May and June, 2000–2014. The Conifer class was measured at Haines Junction Environment Canada station, the Tundra class was measured at Pika Camp station and Icefield was measured at the Divide station. The comparison is for the resampled 1 km NARR grid cells that intersect with the station locations.

**Figure 7:**
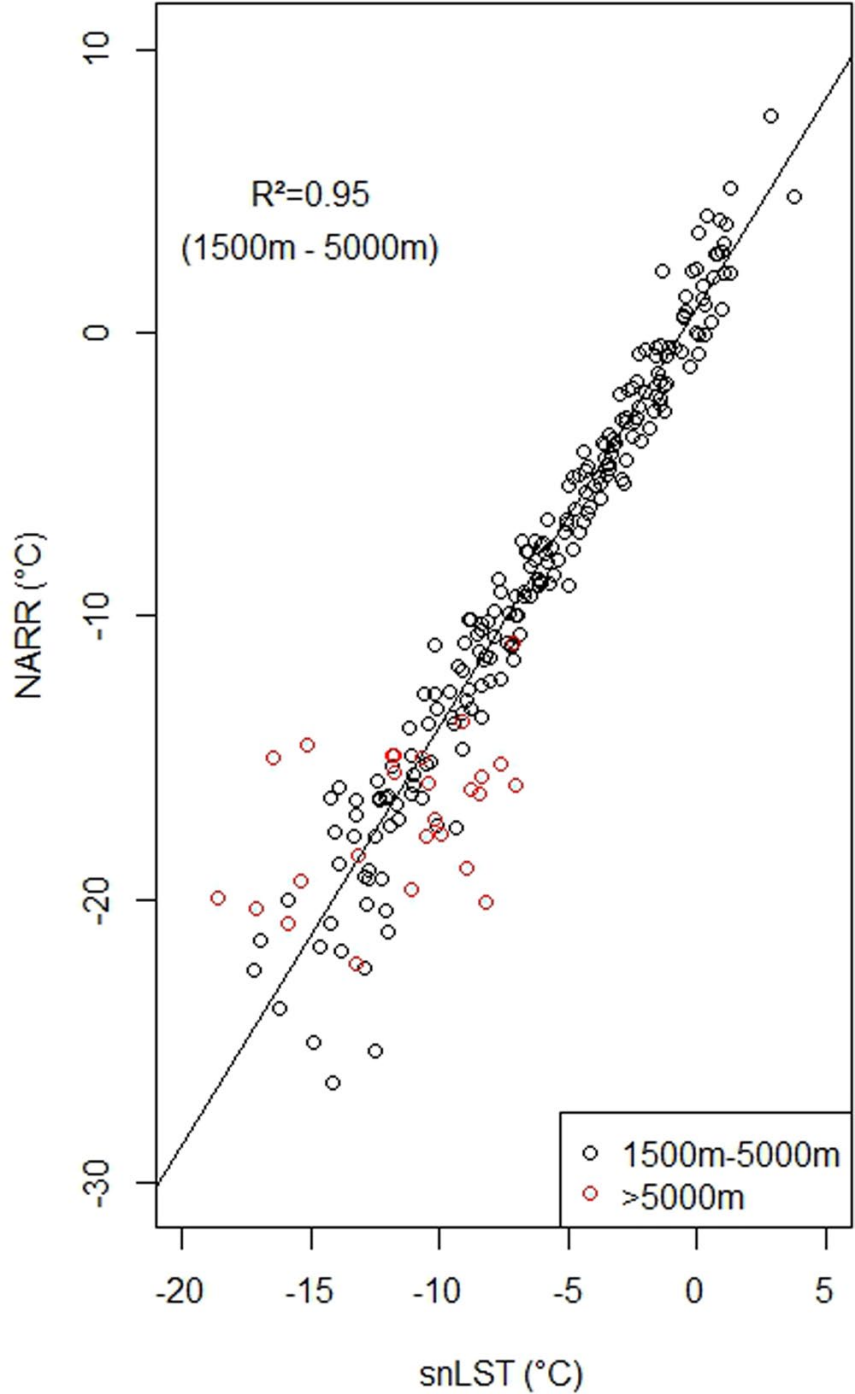
The validation of NARR for high elevation Icefield (almost complete snow cover) using full diurnal average May and June monthly average temperatures. This analysis shows that MODIS LST collected with 2 hours of solar noon (snLST) is unable to validate the NARR at elevations greater than 5,000 m on the Mount Logan massif. The high correlation (R^2^ = 0.95) is calculated for the linear model for data between 1,500 m and 5,000 m (black circles). Red circles are the data originating above 5,000 m. These data display unrealistic behaviour in that some of the NARR values are warmer than the lower elevation NARR data and the solar noon LST provides a temperature range several times larger than at lower elevation.

We used meteorological data and MODIS snLST to validate the NARR temperatures produced for this study. Meteorological station ancillary information is described in the methods section. Figure 6 shows 8-day average downscaled NARR temperatures originating from the three 1 km^2^ grid cells where air temperature is measured in icefield, tundra and conifer forest. The strong correlation (R^2^ = 0.97), despite various amounts of cloud cover and snow cover, provides an indication of the accuracy of the NARR product. However, the highest elevation station is located at 2690 m, leaving the higher elevation NARR grids unvalidated with meteorological data. Instead, we validated the high elevation NARR grid cells with MODIS LST collected within two hours of solar noon for Icefield locations with complete snow cover using the method proposed by Williamson *et al*.^23^ (see Fig. 7). Using 500 m elevation bins, the validation shows that the two variables originating from grid cells below 5000 m have a high correlation (R^2^ = 0.95). Above 5000 m both the NARR and LST values display a high degree of variability and are warmer than the NARR values from originating from the 4500 m to 5000 m elevation bin. The range of solar noon LST values is almost 15 °C above 5000 m. Previous analysis indicates that LST measurements originating from dawn occur much more often than those from solar noon^23^, which indicates that some of the variably displayed in Fig. 6 might be related to data sparsity related to restricting the measurement period to solar noon.

## Discussion

We use a natural experiment and statistical analysis to explore the ability of snow albedo change to drive surface air temperature, instead of calculating snow albedo feedback as a heat flux. We show that downscaled NARR temperature trends during spring are remarkably similar across different land covers with different snow cover trends and variability supporting our hypothesis that external forcing, not snow albedo feedback, is the primary influence on temperature variation. We show that snow albedo decrease causes an infrared surface temperature increase, but this effect appears to be insufficiently large to influence 8-day or monthly average air temperatures. We propose this is because solar noon LST values were collected only for clear sky conditions, which happens only ~25% of days during the month^23^ and because the average air temperature is the full diurnal average. Thus, the influence of the clear sky snLST is dampened in relation to average air temperature under all sky conditions. The temporal trends originate from high spatial resolution grid cells, where the coincident albedo change is interpreted by fractional snow cover, instead of coarse spatial resolution binary snow cover. Our results indicate that temporal and spatial scales are important considerations when calculating the influence of the snow albedo feedback in amplified warming.

We observed a strong elevational dependency in May temperature amplification, which is corroborated by LST collected near solar noon. The trends for change in albedo are increasingly negative with elevation for Tundra and Icefields. The largest negative albedo trends in May occur over complete snow cover at high elevation, and not at lower elevation where the greatest loss of snow extent is occurring. These trends, taken in isolation, suggest that a strong snow albedo feedback is operating in May. The difficulty in measuring the snow albedo feedback is largely related to the difficulty in constraining the amount of temperature and net radiation change directly related to snow cover and snow albedo change. To further complicate this relationship, snow cover extent has been shown to contribute 70–80% of SAF, where the majority of the difference is contributed by crystal growth and aggregation through snow metamorphosis and aging^27^. In June, there was a consistent cooling of 0.09 °C/a across all land cover types and elevations. The cooling trends occur despite large snow cover losses in tundra and albedo declines in both tundra and icefields which are likely related to both snow cover decline and snow aging. Furthermore, the Conifer albedo shows no change over the study period. These lines of evidence suggest that the declines in snow albedo in May and June are most likely the result of an increase in May temperature driven by external forcing, and the influence of the resulting snow albedo feedback on air temperature trends was negligible.

In May, the Icefields daytime cloud fraction changed from positive trends to negative trends above 4,000 m. This reduction in cloud cover does not provide a comprehensive explanation for the basis of the warming trend, which was 0.14 °C/a over the study period, or the elevational dependent portion, which was 0.01 °C/a · 1000 m. This is because between 1,000 m and 2,000 m the daytime cloud cover is increased, and between 2,000 m and 4,000 m the cloud cover trend was essentially zero. The decrease in May daytime cloud cover trends at high elevation is consistent with warming trends, but does not explain the majority of the warming. In June, there was a consistent increase in daytime cloud cover with both land cover class (Conifer-Tundra-Icefield) and elevation which correlates well with the consistent decline in downscaled (NARR) air temperature for this month over the study period. However, May cloud cover trends indicate some control by daytime clouds over temperature trends, which suggests that June cloud cover trends are also responding to external forcing correlated to surface temperature trends.

For many sites, Arctic warming may be strongest at the surface for the majority of the year, but changes in cloud cover have been shown to not influence the warming trend^28^. This warming is coincident with increases in atmospheric water vapour content, which is most likely a consequence of decreasing summer sea ice, a process that has little influence in the southwest Yukon. Currently, it is thought that the majority of solar radiation over the Arctic Ocean is used to melt sea ice and raise the temperature of the ocean surface layer^29^ and thus causes a mild air temperature response and would prove to operate in a fundamentally different way than that of the terrestrial system investigated here.

We present a method to corroborate downscaled air temperature over areas of complete snow cover with MODIS LST collected around solar noon. This technique provides essential temperature validation for high elevation and remote high latitude areas that lack meteorological validation, and increases the number of measurements for these locations by orders of magnitude. The method produces erratic results at the highest elevations for several reasons. The extreme topography of mountain peaks^24^ likely influences the accuracy of both downscaling and satellite thermal skin temperature measurements. The downscaling method used here is limited to the 500 mbar pressure level which should produce a warm bias above ~5,200 m in the native downscaled product, which is the behaviour we summarized in Fig. 7. The summits of tall mountains often have elevation difference of several hundred metres found within 1 km^2^ grid cell^24^. The use of multiple MODIS swaths from Terra and Aqua further expands the temperature range as the complex arrangement of slopes and aspects, and thus surface area, are being imaged from multiple satellite look angles. In addition to the limited amount of LST measurements from high elevation locations, the pausity of LST data originating near solar noon further exacerbates the statistical relationship between air temperature and LST. We limit our analysis of the influence of snow cover and albedo on surface temperature trends to the Conifer and Tundra land covers because these are the areas where the downscaled NARR air temperatures are well validated. Further work is required to reconcile downscaled air temperatures and MODIS LST at the highest elevations in mountain ranges where a large variability in surface aspect and slope occur.

Our results, from a 5,000 m elevational gradient, show temperature fluctuations are primarily responding to external forcing, potentially modulated by day-time cloud cover, which appears to be more influential than the snow albedo feedback. Furthermore, amplified warming occurred high above the surface has also been reported and used to argue for a limited role for snow mediated albedo changes influence Arctic temperature trends^13^. However, both a decreasing trend in poleward heat transport^30^, and increasing poleward heat transport^13^ have been reported indicating that determining the strength of external forcing in the Arctic requires further investigation. Here, May warming rates occur linearly with elevation (i.e., decreasing absolute temperature) which suggests that a temperature feedback might be involved in amplified elevational warming^5^. However, the lack of a corresponding amplified decrease in June cooling with elevation reveals that forcing is not consistent across all elevations, or between months. Furthermore, the SAF which should occur over a short time period (i.e., the change in the length of the melt period) will not be of sufficient strength to modify monthly or seasonal (three month) air temperature, which is typically used for climatological analysis. Our conclusions appear to contradict previous studies of Arctic terrestrial regions that have suggested a much stronger potential for snow-albedo feedback to drive amplified warming than we observed, and our results illustrate the benefits of using elevational gradients and multiple temperature measures to help clarify the impact of different climate forcing mechanisms.

## Summary

While snow albedo and air temperature trends are highly correlated, the snow albedo feedback appears to have a minimal influence on air temperature trends, both at the monthly and 8-day time scales. MODIS snLST indicates the SAF is occurring, but appears to be insufficient to modify average diurnal air temperature. These results suggest that SAF does not scale from discrete, localised events, to coarse temporal and spatial resolutions. May daytime cloud cover trends do not present a coherent explanation for surface temperature trends, suggesting that the correlation with June cooling and cloud cover increase is not a dominant factor in temperature change. A linear elevational amplification in the May warming trend of 0.01 °C/a · 1000 m was found, but in June there is cooling of about 0.09 °C/a uniformly at all elevations, indicating that elevational dependent warming is not consistent over the melt season. The use of solar noon LST to corroborate high elevation May and June average downscaled air temperature trends provides some degree of validation for areas higher than the highest meteorological station in the study area (2,690 m). Although there is some evidence for an albedo dependency in Elevational dependent warming for the Mt Logan region for May, the majority of the warming occurs throughout the study area, regardless of temporal albedo trends, suggesting a large role for external forcing in elevational dependent warming. Our approach for integrating MODIS and NARR observations provides new opportunities for untangling the complex climate feedback processes in snow covered terrain in mountain and polar environments.

## Methods

Downscaled North American Regional Reanalysis (NARR) and MODerate resolution Imaging Spectroradiometer (MODIS) satellite imagery of Land Surface Temperature (LST), Fractional Snow Cover, Cloud Cover, Broadband Black Sky Albedo (over vegetated surfaces) and Snow albedo (over permanent snow and ice) were aggregated into May and June averages from the standard 8-day MODIS composite range. Monthly averages for MODIS data are composed of four 8-day periods that start on non-leap year Day of Year 121 and correspond to the MODIS albedo 8-day periods, thus the May average encompasses Day of Year (DoY) 121–152, and June encompasses DoY 153–184. Monthly averages for meteorological data conform to the calendar month. All of the spatial layers were projected to Albers Equal Area and were resampled to a spatially co-ordinated grid system of 1 km grid cells, in the WGS 84 datum.

### MODIS LST

The MODIS LST data used in this study are clear-sky reprocessing version 5 Terra and Aqua swath data (MOD11_L2 & MYD11_L2), gridded at 1 km^31,32^. MODIS LST is derived from surface infrared emission and a global emissivity look-up table^33^, and is often referred to as skin temperature. MODIS data were downloaded from the Land Processes Distributed Active Archive Center – LPDAAC. The daytime MODIS LST product cloud mask has a larger confidence in identifying contamination than the night-time mask^34^. Previous work in the southwest Yukon has shown cloud contaminates <13–17% of daytime MODIS LST values, with a maximum temperature depression of 2 K^35^. Averaged over a month cloud, contamination will reduce the average solar noon LST by less than 1 K.

### Solar noon LST

Solar noon LST (snLST) was determined by identifying MODIS Aqua and Terra swaths representing overpasses occurring within 2 h before and after solar noon at 61°N and 139.5°W (which is the approximate centre of the study area), and then selecting the maximum value from 2 to 4 daily images. This method exploits the maximum overpass density, which occurs around solar noon, to produce a near-noon temperature product. We use daytime LST because it is most sensitive to SAF; albedo decreases related to snow melt should correlate most strongly with daytime maximum temperatures, rather than minimum temperatures^36^. Minimum LST values were not calculated because minimum LST (collected at night) skews average LST to low values compared to average air temperature^37,38^. With only one meteorological station within the Icefield to validate high elevation reanalysis data, MODIS LST was used to corroborate NARR trends. Previous analysis^23^ showed that NARR average temperature is highly correlated with LST collected within 2 hours of solar noon for high snow cover fractions.

### MODIS albedo

MODIS albedo used in this study originated from two distinct albedo products – the MCD43A3 product, which was used for vegetated land cover, and the MOD10A1, which was used for snow covered permanent snow and ice. Vegetation albedo measurements (MCD43A3) are acquired from the MODIS sensor on the Aqua and Terra satellites, which are gridded at 500 m in 8-day composites. This product uses the Bidirectional Reflectance Distribution Function (BRDF) and MCD43A1 albedo model parameter product^39,40^ and the MODIS cloud mask to produce White-sky and Black-sky albedos. White-sky albedo is bihemispherical reflectance under isotropic illumination conditions and has the angular dependency eliminated. Black-sky albedo is the directional hemispherical reflectance, which is produced for local solar noon. The albedo product is either the full inversion BRDF, or when insufficient observations are available the magnitude inversion is produced. The magnitude inversion uses an *a priori* knowledge backup algorithm is used to produce the magnitude inversion albedo values, which performs well in the majority of cases^41,42^. We use both the full inversion and magnitude inversion for the MCD43A3 product, which was informed by field validation studies for the Kluane study area over a wide range of alpine land cover types^43^. Key findings from the validation exercise indicated (i) the magnitude inversion albedo measurements are produced approximately 10 times more frequently than the full inversion, (ii) black sky and white sky albedo products are nearly identical when compared to field albedo measurements, and (iii) the MCD43A3 product produced far fewer albedo measurements over snow and ice than the MOD10A1 snow albedo product. Therefore we used the MOD10A1 snow albedo product for areas classified as Icefields and used the MCD43A3 product for the lower elevation land covers. The daily MOD10A1 snow albedo produced from the MODIS sensor on the Terra satellite^44^ was produced for cloud-free conditions. The MOD10A1 snow albedo is produced from the highest scoring single daily observation, based on an algorithm that ingests illumination and view angles, in addition to cloud mask and fractional snow cover (MOD10A1 User Guide: http://modis-snow-ice.gsfc.nasa.gov/uploads/sug_c5.pdf) as an input.

### MODIS fractional snow & daytime cloud cover

The snow cover product used in this study is the MODIS Terra daily fractional snow cover product produced at 500 m resolution determined through regression equations of normalised difference snow index (NDSI) and fractional snow cover^45^. MODIS provides a measure of snow cover using an algorithm based on the NDSI, the normalised difference vegetation index (NDVI) for forested areas, a thermal mask and a cloud mask^44^. The daily fractional snow cover was resampled to 1 km grid cells using an arithmetic average of the 4 possible values, ignoring values other than 0–100%. The daily maps of 1 km snow cover were aggregated to eight-day averages showing a value of 0–100% for each aggregated grid cell.

Grid cells in the snow cover layers that were identified by the MODIS cloud mask as cloud covered were used to produce a binary classification of daytime cloud cover (cloud covered or clear), after which these 500 m grid cells were arithmetically averaged to 1 km. Cloud cover is daytime cloud cover because it is extracted from the snow cover product, which incorporates the visible portion of the electromagnetic spectrum. The purpose of using daytime cloud cover is to identify variation in incoming solar radiation, which would influence net radiation independently of albedo.

### NCEP North American Regional Reanalysis (NARR) downscaling

A mix of statistical and dynamical downscaling methods were applied to atmospheric temperatures from North American Regional Reanalysis (NARR) for 16 pressure levels between 1000 mbar and 500 mbar air temperature to produce the downscaled temperature product used in this study. NARR is the National Center for Environmental Prediction (NCEP) product that uses surface, radiosonde, and satellite data combined in the Eta forecasting system^46^. The downscaling method uses a three-part, piece-wise fitting to NARR vertical air profiles and subsequent interpolation of those fitted parameters to a 200 m grid for prediction of temperature at arbitrary elevation, while using an interpolation scheme that reconstructs vertical temperature profiles from a regional reanalysis and does not require meteorological station measurements for tuning^47^. This method was developed for western Canada with the aim of producing temperature fields that are more accurate than other downscaled products for high elevation, high vertical relief glaciated areas. Validation against 2 m air temperature measured at 78 stations in southern British Columbia showed a mean bias of 0.5 °C for areas with high vertical relief, from the data set encompassing 1990 to 2008^47^. A mean absolute error of no more than 2 °C for monthly averages was also reported^47^. The native 200 m downscaled NARR grid cells above ~5,200 m likely have pressure levels below 500 mbar, which should lead to warm bias above this elevation. The daily averaged downscaled NARR were resampled to 1 km grids using cubic convolution and averaged to monthly time periods. The 1 km grid size corresponds to the MODIS LST grid and is high enough spatial resolution to identify elevational temperature dependency.

### Meteorological Observations

Meteorological observations of air temperature and snow cover observations originated from three locations within the study area. Although there are two Environment Canada meteorological stations within the study area (Burwash Landing and Haines Junction) we could not confirm whether the Burwash Landing station was used in the production of NARR data. Therefore we use only data provided from the Haines Junction Station. Snow cover observations are recorded at the Haines Junction Environment Canada stations. Air temperature measurements were made at the Haines Junction Environment Canada station (in the conifer land cover), the Divide station (on the snow covered icefield) east of Mount Logan, and at Pika Camp (alpine tundra). Validation of the NARR downscaled temperature product for the three land cover types uses data from the Haines Junction, Divide and Pika Camp stations. The snow cover amounts for the three validation stations ranged from no snow (Haines Junction), variable snow cover (Pika Camp), and nearly complete snow cover (Divide). Meteorological measurements are sparse in the southwest Yukon, especially away from the Alaska Highway. Haines Junction data were from 2000–2014, Pika Camp data were from 2007–2014, the Divide station data were from 2010–2014 (Fig. 1). The Haines Junction station is located at 60.77°N; 137.58°W and at an elevation of 599 m and in the Conifer class. The Pika Camp station is located at 61.21°N; 138.28°W (1,635 m) in sparsely vegetated Tundra, and the Divide station is located at 60.70°N; 139.81°W (2,690 m) in permanent snow covered Icefield.

### Statistical analysis

Linear slopes, standard errors and p-values were calculated using the R software package (version 3.4.3) using the linear model and summary functions on the non-aggregated data sets. All spatial data manipulation was conducted with ArcGIS (version 10.2.1).

## Electronic supplementary material

Dataset 1
