# Supplementary material for: Spring warming in Yukon mountains is not amplified by the snow albedo feedback
Source: Sci Rep. 2018 Jun 13;8:9000. doi: 10.1038/s41598-018-27348-7 (PMC5998050; doi:10.1038/s41598-018-27348-7)
Supplement: Supplementary file 1 — Dataset 1 [file 41598_2018_27348_MOESM1_ESM.docx]

Spring warming in Yukon mountains is not amplified by the snow albedo feedback

Scott N. Williamson^1,2*^, Faron S. Anslow^3^, Garry K. C. Clarke^4^, John A. Gamon^1,2^, Alexander H. Jarosch^5^, David S. Hik^1^

^1^Department of Biological Sciences, University of Alberta, Edmonton, Alberta, Canada T6G 2E9

^2^Department of Earth and Atmospheric Sciences, University of Alberta, Edmonton, Alberta, Canada T6G 2E3

^3^Pacific Climate Impacts Consortium, University of Victoria, Victoria, British Columbia, Canada V8W 3R4

^4^Department of Earth, Ocean and Atmospheric Sciences, University of British Columbia, Vancouver, Canada, V6T 1Z4

^5^Institute of Earth Sciences, University of Iceland, Sturlugata 7, 101 Reykjavik, Iceland

*Corresponding author. Tel.: +1 (780) 492-4863, E-mail address: snw@ualberta.ca

Supplemental Table 1: May Mean and Standard Errors (se) for downscaled North American Regional Reanalysis (narr) and MODIS solar noon LST (snLST), snow fraction (sf), black sky albedo (bsa), white sky albedo (wsa), daytime cloud fraction (cf) and snow albedo (sa), which correspond to Figure 2 (a).

Supplemental Table 2: June Mean and Standard Errors (se) for downscaled North American Regional Reanalysis (narr) and MODIS solar noon LST (snLST), snow fraction (sf), black sky albedo (bsa), white sky albedo (wsa), daytime cloud fraction (cf) and snow albedo (sa) , which correspond to Figure 2 (b).
